# Assessing Short-Video Dependence for e-Mental Health: Development and Validation Study of the Short-Video Dependence Scale

**DOI:** 10.2196/66341

**Published:** 2025-03-04

**Authors:** AnHang Jiang, Shuang Li, HuaBin Wang, HaoSen Ni, HongAn Chen, JunHong Dai, XueFeng Xu, Mei Li, Guang-Heng Dong

**Affiliations:** 1 Zhejiang Key Laboratory for Research in Assessment of Cognitive Impairments Hangzhou China; 2 Center for Cognition and Brain Disorders Affiliated Hospital of Hangzhou Normal University Hangzhou China; 3 Department of Psychology Yunnan Normal University Kunming China

**Keywords:** short-video dependence, problematic short-video use, cutoff point, scale development, mental health, short video, internet addiction, latent profile analysis, exploratory factor analysis, confirmatory factor analysis

## Abstract

**Background:**

Short-video dependence (SVD) has become a significant mental health issue around the world. The lack of scientific tools to assess SVD hampers further advancement in this area.

**Objective:**

This study aims to develop and validate a scientific tool to measure SVD levels, ensuring a scientifically determined cutoff point.

**Methods:**

We initially interviewed 115 highly engaged short-video users aged 15 to 63 years. Based on the summary of the interview and references to the Diagnostic and Statistical Manual of Mental Disorders, Fifth Edition (DSM-5) criteria for behavioral addictions, we proposed the first version of the short-video dependence scale (SVDS). We then screened the items through item analysis (second version) and extracted common factors using exploratory factor analysis (third version) and confirmatory factor analysis (final version). Convergent validity was tested with other scales (Chinese Internet Addiction Scale [CIAS] and DSM-5). Finally, we tested the validity of the final version in 16,038 subjects and set the diagnostic cutoff point through latent profile analysis and receiver operating characteristic curve analysis.

**Results:**

The final version of the SVDS contained 20 items and 4 dimensions, which showed strong structural validity (Kaiser-Meyer-Olkin value=0.94) and internal consistency (Cronbach α=.93), and good convergent validity (*r*_CIAS_=0.61 and *r*_DSM-5_=0.68), sensitivity (0.77, 0.83, 0.87, and 0.62 for each of the 4 dimensions), and specificity (0.75, 0.87, 0.80, and 0.79 for each of the 4 dimensions). Additionally, an SVDS score of 58 was determined as the best cutoff score, and latent profile analysis identified a 5-class model for SVD.

**Conclusions:**

We developed a tool to measure SVD levels and established a threshold to differentiate dependent users from highly engaged nondependent users. The findings provide opportunities for further research on the impacts of short-video use.

## Introduction

Short videos are immensely popular digital content, with platforms like TikTok allowing users to create and share videos ranging from a few seconds to a few minutes [1]. These apps engage users by facilitating viewing, creation, and commenting, changing self-presentation and cultural learning. In 2022, TikTok had 1.2 billion monthly active users, with an average viewing time of 168 minutes per day [2]. Hanaysha [3] noted that during the COVID-19 pandemic, short-video apps became key sources of news and entertainment. These apps have impacted various aspects of the world, such as popular culture, content creation and consumption, and the spread of information on political and social issues.

Short-video apps also raised public concerns about their potential negative consequences, such as distraction, poor time management, and mental health problems [1,4,5]. A number of studies have been conducted in this area. For instance, Domoff et al [6] developed a scale to measure the extent of problematic media use considering the need. However, the lack of reliable and valid tools for assessing the corresponding qualities of short-video usage has hindered the progress of research in this field.

Before developing a scale or questionnaire for short-video dependence (SVD), we need to justify not using the term “addiction,” which requires careful consideration to establish standards. First, the latest version (fifth edition) of the Diagnostic and Statistical Manual of Mental Disorders (DSM-5) recognizes internet gaming disorder (IGD) as a provisional disorder [7], but not short-video disorder. Second, internet-related disorders are often more about specific online activities than excessive internet use [7,8]. Third, some studies have suggested that short-video users show more “dependence” than “addiction,” with behavior being more habitual than pathological [9]. Thus, “dependence” is a more appropriate term in this context.

Some researchers have tried to develop internet-related behavioral disorder scales but have faced limitations pertaining to measuring SVD. Güzeller and Coşguner [10] developed a problematic cell phone use scale for Turkish adolescents considering the need for a measurement tool on problematic cell phone use in this population. In addition, these articles were published early, before TikTok was available, and have less applicability to the present context. Domoff et al [6] developed a tool for assessing screen media addiction in children aged 4 to 11 years. The results of the study indicated that the scale has good psychometric properties in terms of factor structure, reliability, and convergent validity for the measurement of problematic media use in children younger than 12 years. However, the limitations of the study include a lack of methodological constraints in assessing screen time and insufficient racial or ethnic diversity of the sample.

The short-video dependence scale (SVDS) was established based on the IGD diagnostic criteria from the DSM-5, since internet addiction has most convincingly been linked to short-video use [11,12], and research addressing dependence behavior is lacking as compared to research on addiction. The DSM-5 is a widely accepted and used classification system for mental disorders that is used to diagnose and categorize mental health problems. It provides standardized definitions and classifications for a variety of mental disorders, including IGD, gambling disorder, etc. According to the DSM-5, a clinical diagnosis of IGD is indicated if 5 or more of the following 9 criteria are met: preoccupation, withdrawal, tolerance, loss of control, loss of interest, continuation, deception, escape, and jeopardizing. By using the DSM-5 as its developmental framework, the SVDS aims to align with the diagnostic criteria and characteristics of IGD, thereby ensuring the validity and comparability of measurements.

Therefore, the DSM-5 criteria for IGD have some advantages in identifying and assessing SVD, but these are not fully applicable or comprehensive. First, SVD is similar to IGD in many ways, such as excessive use, uncontrollable desire, and interference with daily functioning. However, some criteria may not be suitable or sufficient for short-video addiction, such as tolerance, loss of interest, and jeopardizing. For instance, loss of interest may not encompass the full spectrum of motivational factors that affect short-video addiction, such as social comparison, self-presentation, and peer pressure [1]. Furthermore, users may still be able to carry out their daily activities even after spending an excessive amount of time watching short videos, and thus, jeopardizing may not be as severe or uncommon with regard to SVD. Neglecting or compromising other significant facets of life, like relationships, career, or health, might be a more appropriate criterion. The DSM-5 diagnostic criteria for IGD will be improved and refined by the development of the SVDS, which will include extra items identified through in-depth interviews.

Most importantly, to determine the cutoff point for the SVDS among a large sex-balanced sample of Chinese young adults, latent profile analysis (LPA) was used, which is an epidemiological approach to determine the cutoff point when a clinical interview is not available [13-15]. It allows for the identification of distinct groups of short-video users based on their responses in the SVDS, with the group exhibiting the highest levels of SVD being identified as “dependent short-video users” for determining the optimal cutoff point. To date, numerous studies have applied the LPA approach to derive the optimal cutoff points for measurement tools, such as the Bergen Social Media Addiction Scale [16], Online Social Networking Addiction Scale [13], Dimensional Anhedonia Rating Scale [17], and Smartphone Application-Based Addiction Scale [14].

In summary, the field of SVD lacks standardized definitions and measurements for short-video addiction, which may hinder the comparability and generalizability of the results and further hamper the study of its prevalence, impeding crucial future research in the field of SVD. The evolving internet landscape demands a contemporary scale. This study aims to develop and validate a specific SVDS for SVD, drawing on established frameworks and diagnostic criteria from IGD research. The SVDS provides a standardized tool with a clear diagnostic cutoff point to assess SVD and understand its prevalence, correlates, and consequences.

## Methods

### Ethical Considerations

The experiment conforms to the Code of Ethics of the World Medical Association (Declaration of Helsinki). The Human Investigations Committee of Hangzhou Normal University approved this research (approval number: 20190505). All participants provided written informed consent before experimentation.

### Participants

From October 2022 to December 2023, 4 surveys were conducted online, among which, the first 3 surveys employed 2 data collection platforms in China, namely the TClab platform and NAODAO platform. The last survey involved 16,038 participants from Hangzhou Normal University and Zhejiang University of Water Resources and Electric Power, resulting in a total of 17,266 Chinese participants (Table 1; Multimedia Appendix 1). To clarify, participants were recruited using convenience sampling, and all data collection was conducted virtually.

**Table 1:** Demographic information of the participants for 4 tests.

Version	Additional scales	Valid sample, N	Age (years), mean (SD)	Age (years), minimum	Age (years), maximum	Female percentage
Pretest	—^a^	457	23.67 (6.84)	16	57	39%
SVDS^b^ 1.0-3.0	CIAS^c^ and DSM-5^d^	402	24.94 (7.21)	17	45	44%
SVDS 4.0	CIAS and DSM-5	369	27.05 (8.18)	17	63	46%
SVDS 4.0	DSM-5	16,038	19.86 (2.14)	15	41	48%

^a^Not applicable.

^b^SVDS: short-video dependence scale.

^c^CIAS: Chinese Internet Addiction Scale.

^d^DSM-5: Diagnostic and Statistical Manual of Mental Disorders, Fifth Edition.

### Methodological Overview of the Entire Study

**Figure 1:**
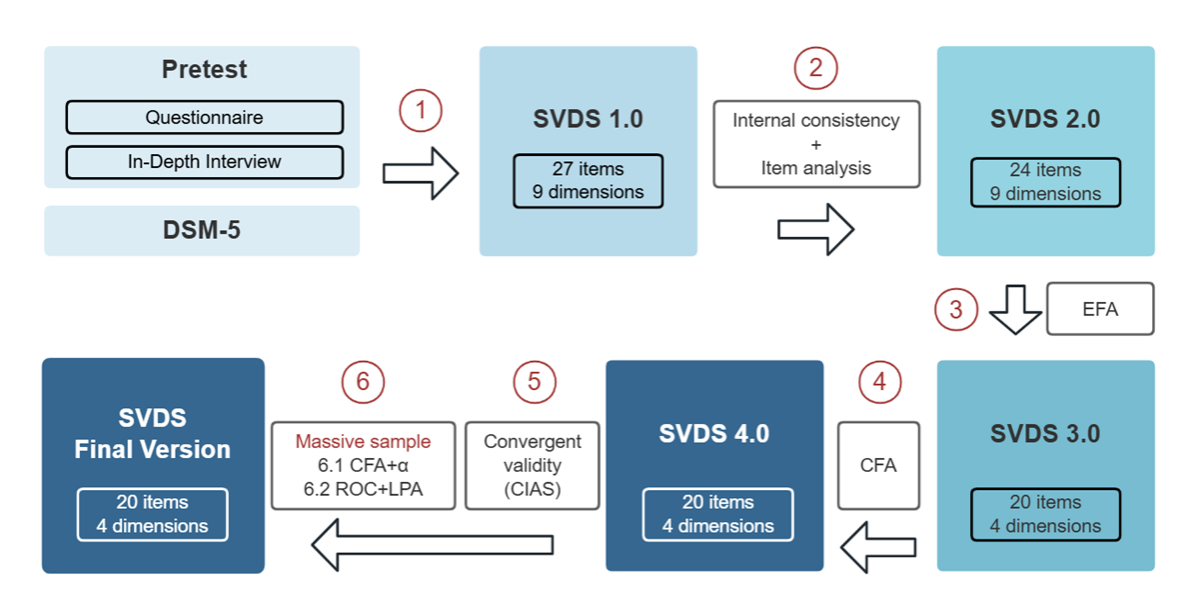
Methodological overview of the entire study. CFA: confirmatory factor analysis; CIAS: Chinese Internet Addiction Scale; DSM-5: Diagnostic and Statistical Manual of Mental Disorders, Fifth Edition; EFA: exploratory factor analysis; LPA: latent profile analysis; ROC: receiver operating characteristic; SVDS: short-video dependence scale.

Figure 1 shows the complete process of developing the SVDS. There were several stages. First, in the preparation stage, we screened out people with a high degree of SVD through the questionnaire, obtained the dependence symptoms of these people through interviews, and then combined the findings with the DSM-5 to prepare the first version of the SVDS. Second, SVDS 2.0 was created after internal consistency was examined and item analysis was conducted. Third, through exploratory factor analysis (EFA), we removed the unqualified questions and obtained the third version of the SVDS. Fourth, after confirmatory factor analysis (CFA) and another round of screening, the fourth version was established. Finally, we tested the convergent validity, conducted another round of CFA with a massive sample, and confirmed the cutoff points using receiver operating characteristic (ROC) curve analysis and LPA.

### In-Depth Interviews

The in-depth interview method is a fundamental psychological research approach that involves face-to-face conversations between interviewers and participants to understand the psychological and behavioral aspects of the participants [18]. The method is applied in various disciplines; in-depth interviews are also conducted in the form of online conversations [19,20]. As a qualitative research method, in-depth interviews are effective in promptly identifying issues and complementing the questionnaire survey method. In this study, the in-depth interview method was used in conjunction with the questionnaire survey. The process of data collection specifically involved preliminary screening of the pretest questionnaire. The screening criteria were as follows: (1) daily viewing of more than 4 hours, (2) significant impact of short-video viewing on daily life, and (3) voluntary acceptance of the interview and recording. Consequently, semistructured interviews were conducted with 15 short-video users of different ages, users from different regions, and users with different usage levels (Multimedia Appendix 2). The interview content was analyzed using the qualitative analysis software NVivo 12 (Lumivero). The key concepts were derived from the interviews [19], and referring to the 9 criteria of the DSM-5, the initial version of the SVDS with 27 items was developed.

The in-depth interview questions in this study were divided into 5 categories: basic questions, short-video platform usage and dependence, in-depth questions, suggestions and countermeasures, and self-assessment questions with ratings (Multimedia Appendix 3). The main aim was to understand the participants’ motivations for using short-video platforms and their levels of dependence and to gain a deeper understanding of the specific symptoms of strong dependence. During the interviews, the interviewer focused on the purpose, motivations, experiences, and dependence of short-video platforms in each round of the interviews. Subsequently, the interview content was coded to determine the dimensions and specific items for the first version of the SVDS. In terms of dimensions, small-scale screening was conducted based on the 9 addiction features of the DSM-5, resulting in the identification of 9 dimensions and 27 items for the SVDS. Moreover, to clarify, we used random sampling to recruit participants. The participants were collected through the NAODAO and TClab platforms.

### Encoding In-Depth Interview Data According to the Classical Grounded Theory

The grounded theory establishes a close connection between empirical research and theory construction, enabling researchers to develop concepts and construct theories by systematically analyzing and summarizing empirical data [21,22]. The use of in-depth interviews in this study generates a substantial amount of textual data. By applying the grounded theory, specifically open coding, and aligning it with the DSM-5 scale, key conceptual keywords are abstracted, allowing for a direct reflection of the key points covered in the interview content. This process aids in determining the main dimensions upon which the questionnaire design should be based. The sample saturation point was achieved after the completion of the 15th in-depth interview for this study.

### Establishment of the First Version of the SVDS

The first version of the SVDS consisted of 27 items (Multimedia Appendix 4). As for the dimensions, the IGD scale from the DSM-5 consisted of 9 dimensions: preoccupation, tolerance, withdrawal, persistence, escape, problems, deception, displacement, and conflict. These 9 dimensions encompassed most of the situations reported by users with a high dependency on short videos, so we used them as a basis for expansion. Meanwhile, items from multiple related scales were collected for the SVDS question bank for further use [7,23-31]. Additionally, we modified the 9 dimensions by considering the actual situation of users who were highly dependent on short videos in the initial screening. Specifically, we counted the frequency of each dimension mentioned in the narratives of highly dependent users of short videos and removed dimensions that were mentioned zero times. Furthermore, a new dimension, “lie detection,” was added. As for the items, we developed 3 items for each dimension based on the information coded in the in-depth interviews, which resulted in the first version of the SVDS.

### Developing the Second Version of the SVDS After Conducting Item Analysis

Prior to the EFA, we conducted item analysis [32,33] in sample 2 (n=402) and sample 3 (n=369), which is the process of assessing the quality and effectiveness of each item in the scale. Item analysis mainly includes indicators such as item difficulty, item differentiation, and item relevance. Initially, we removed unqualified samples, which were first preprocessed according to the following two steps: (1) removing samples with missing values and manually removing samples where the length of time was shorter than 2 SDs from the mean and all answers were the same and (2) converting the reversed items to numerical values, recording and scoring them accordingly, and assigning new values to the original items. For further information, sample 2 and sample 3 were also recruited using random sampling. They were primarily gathered through the NAODAO and TClab platforms.

We then screened the items according to 2 indicators during item analysis. The first indicator was the correlation between each item’s score and the total score. Based on the Pearson correlation coefficient, the deletion criteria were as follows: (1) nonsignificant correlation between the items and the total scale (*P*>.05) and (2) correlation coefficient (*r*) between the item score and the total score below 0.45. The other indicator was the reliability coefficient, and the criteria for deletion were as follows: (1) correlation coefficient between the modified item and the total score lower than 0.45 and (2) increase in the internal consistency coefficient (Cronbach α) after item deletion. This resulted in the second version of the SVDS (Multimedia Appendix 5).

### Developing the Third Version of the SVDS After Conducting EFA

The suitability of the items of the first version of the SVDS for factor analysis was assessed using the Kaiser-Meyer-Olkin (KMO) value and Bartlett Test of Sphericity. Additionally, principal component analysis (PCA) with varimax rotation was conducted on the 21 items of the first version of the SVDS. Items that did not meet the established standards and theoretical expectations were removed. The deletion criteria were as follows: (1) measures of sampling adequacy (MSA) below 0.8, (2) typical values below 0.3, (3) factor loading lower than 0.45, (4) items appearing in two or more factors simultaneously, and (5) factors containing only 1-2 items.

The remaining items were subjected to EFA to obtain the new dimensions of the SVDS based on the factor eigenvalues. This resulted in the third version of the SVDS (Multimedia Appendix 6).

### Developing the Fourth Version of the SVDS After Conducting CFA

CFA was conducted to assess the goodness of fit between the proposed model and the collected data [34,35]. Based on the factor solution obtained from the EFA, a model diagram was constructed. CFA was then performed using AMOS24, and model fit was evaluated using the comparative fit index (CFI), Tucker-Lewis Index (TLI), normed fit index (NFI), iterative fit index (IFI), root mean square error of approximation (RMSEA), and 90% CI. When dealing with large samples, the CFI and RMSEA are considered informative fit criteria in structural equation modeling [36]. A good fit is indicated when the CFI, TLI, NFI, and IFI are all greater than 0.9 and the RMSEA is less than 0.08 [34,37].

### Convergent Validity and Reliability of the Fourth Version of the SVDS

Convergent validity was determined by examining the correlations between the SVDS scores and the frequency of daily short-video platform use, as well as the DSM-5 and Chinese Internet Addiction Scale (CIAS) scores [24,38,39]. The CIAS is a 19-item self-report scale, with each item rated on a scale of 1-4 (1=completely disagree, 2=somewhat disagree, 3=somewhat agree, and 4=completely agree). The total score ranges from 0 to 76. Participants with a score of 53 or above were classified as having internet addiction. The CIAS has been shown to have satisfactory reliability and validity in the Chinese population [39,40]. In the case of good convergent validity, we expected a strong correlation between SVDS and CIAS scores. Additionally, the internal consistency (reliability) of the scales was assessed using Cronbach α in the 2 sample datasets.

### Use of Large Samples to Confirm the Reliability and Validity of the Fourth Version

To assess the final version of the SVDS, we applied it to 16,038 subjects, conducted a CFA to test the validity, and tested the reliability using Cronbach α. The test criteria for the indicators were the same as above. For further clarification, participants in sample 4 were drawn from all first-year students at a college in Hangzhou.

### Establishment of Cutoff Points for Dependence and Nondependence

To determine the cutoff point for the population and identify individuals with the disorder, we used the DSM-5 criteria. The group of dependent short-video users according to the DSM-5 (getting more than 5 points out of 9) was considered the “gold standard.” Furthermore, ROC curve analysis was adopted to determine the optimal clinical cutoff point based on the gold standard for clinical diagnosis. The diagnostic efficacy of the Bergen Social Media Addiction Scale was assessed through the area under the ROC curve (AUC). Sensitivity analysis was adopted to determine the optimal clinical cutoff point for the SVDS. The sensitivity (the proportion of true positives), specificity (the proportion of true negatives), 95% CI, positive prediction rate (PPR; the proportion of correctly diagnosed positive cases), and negative prediction rate (NPR; the proportion of correctly diagnosed negative cases) were calculated.

LPA was then conducted in the community sample to identify the groups of adolescents with a higher risk of being addicted to short videos among dependent short-video users in MedCalc Version 22.01 [13,14]. Models with 2-6 latent profiles were estimated based on the scores of the 20 items of the SVDS. The Akaike information criterion (AIC), Bayesian information criterion (BIC), sample size–adjusted Bayesian information criterion (SSABIC), and entropy of each model were examined. Finally, the Lo-Mendell-Rubin adjusted likelihood ratio test (LMRT) was used to determine the best-class solution. Considering the probabilistic nature of the LPA classes, the Wald chi-square test of mean equality for latent class predictors in mixture modeling was used for these comparisons.

## Results

### Coding of In-Depth Interview Data to Derive the First Version of the SVDS

In the preliminary survey, we conducted in-depth interviews with 15 participants who were identified as high-dependence users of short videos from a pool of 457 participants. Subsequently, the interview data were subjected to open coding, taking into account the 9 characteristics from the DSM-5 criteria (Multimedia Appendix 7). This process resulted in the identification of 9 dimensions and 27 specific items for the first version of the SVDS (Multimedia Appendix 4). Open coding focuses on assigning relevant concepts to the data, enabling researchers to categorize the information more clearly. In this study, to present the interview content of the participants more coherently, all original data were systematically numbered and sorted. The in-depth interview data, serving as the primary source of information, were labeled as M1, M2, F3, M4, M5, M5, M7, F8, M9, M10, M11, M12, M13, F14, and M15 (Multimedia Appendix 2). While comprehensive open coding was performed on the primary data, other materials were used for supplementary reference. By conducting open coding on the interview data of the 15 participants, the dimensions and specific items for the first version of the SVDS were determined. In the high-dependence group of short-video users, the most frequently occurring feature was “obsession,” while the dimension of “conflict” did not appear at all. Therefore, we eliminated the “conflict” dimension. Considering that the DSM-5 scale consists of only 9 items while the SVDS includes 24, we deemed it necessary to add a dimension related to deception (comprising 3 items) to screen for valid questionnaires.

### Item Analysis of the First Version of the SVDS

Before conducting the EFA, a preliminary item analysis [32,33] was performed. First, 402 samples were left after excluding the unqualified samples. Next, after excluding the polygraph item, item analysis was carried out on a total of 24 items. Initially, the correlation between each item’s score and the total score was calculated prior to item screening. Based on the Pearson correlation coefficient, the analysis revealed that items 4 (*r*=0.43; *P*=.23), 15 (*r*=0.32; *P*=.50), 16 (*r*=0.14; *P*=.33), and 24 (*r*=−0.19; *P*=.55) did not meet the established criteria, resulting in their deletion.

Furthermore, the reliability coefficient was used for item selection. The internal consistency α coefficient of the 24 items was found to be .90, indicating satisfactory internal consistency of the scale. The results indicated that the correlation coefficients of the modified items in items 15 (*r*=0.34; α=.90), 16 (*r*=0.16; α=.91), and 24 (*r*=0.33; α=.90) were all below 0.4. Thus, the 3 items that fulfilled the condition for an increase in the internal consistency coefficient (α) were retained and considered deleted. Consequently, combined with the above item analysis methods, 3 items (15, 16, and 24) were deleted and 24 items were retained, and thus, the second version of the SVDS was obtained (Multimedia Appendix 5).

### EFA of the Second Version of the SVDS and the Establishment of 4 Dimensions

The suitability of the items of the second version of the SVDS for factor analysis was assessed using the KMO value (KMO=0.94) and Bartlett Test of Sphericity (*χ^2^*_325_=7692.17; *P*<.001). Additionally, the polygraph item analysis showed that higher scores on the items indicated participants’ failure to answer the questions honestly [41]. For our study, 402 valid sample groups were retained in the second sample and 369 valid sample groups were retained in the third sample, meeting the minimum requirement for the number of observations per variable [42].

PCA with varimax rotation was conducted on the 21 items of the second version of the SVDS. Items that did not meet the established standards and theoretical expectations (item 4) were removed. Specifically, the factor loading of item 4 was 0.38 (*P*<.001), which was the lowest among all items and was below 0.4.

After deleting 4 items, 3 items were not needed for lie detection. The remaining 20 items were subjected to EFA, resulting in the emergence of 4 factors with eigenvalues greater than 1, explaining a cumulative variance of 62.5% (Multimedia Appendix 8). All items demonstrated commonalities ranging from 0.50 to 0.70 and factor loadings ranging from 0.48 to 0.78. Factor 1, named “withdrawal and tolerance,” consisted of 5 items (5, 3, 2, 6, and 1), which referred to symptoms of maladaptation following cessation of short-video watching and reduced effects due to repeated or prolonged use. Factor 2, named “preoccupation and persistence,” comprised 5 items (8, 7, 10, 12, and 11), reflecting persistent thoughts and behaviors related to short-video use. Factor 3, named “damage and hindrance,” encompassed 5 items (9, 20, 21, 18, and 17), indicating the negative impact on physical and mental health, which hindered personal growth and development. Factor 4, named “deception and escape,” consisted of 5 items (13, 24, 22, 14, and 23), signifying the concealment of short-video use from others and using short videos as a means of avoiding problems. Consequently, the third version of the SVDS comprised 20 retained items (Multimedia Appendix 6).

### CFA and the Internal Consistency of the Third Version of the SVDS

CFA was conducted to assess the goodness of fit between the proposed model and the collected data, and model fit indices were employed. In the evaluation of the goodness of fit, CFI, NFI, IFI, and TLI values should exceed 0.90, while RMSEA should be less than 0.08 for a satisfactory model fit [43]. The results of the goodness-of-fit analysis were as follows: *χ^2^*/df=2.366; RMSEA=0.058; CFI=0.943; NFI=0.906; IFI=0.943; and TLI=0.921. All the data met the acceptable fit index criteria. Moreover, the internal consistency α coefficient of the 20 items was found to be .91. Therefore, the fourth version of the SVDS was identical to the third version (Multimedia Appendix 9).

### Convergent Validity of the Final (Fourth) Version of the SVDS

To assess the convergent validity, the scores of the respondents in the first version and the final version of the SVDS were correlated with their self-declared SVD and related constructs, such as the frequency of daily short-video platform use, DSM-5 scores, and CIAS scores. The DSM-5 scale refers to the Short Video Addiction Scale, which is a simple adaptation of the Internet Addiction Scale. As shown in Table 2, all correlations were significant at *P*<.001 and aligned with the expected directions. Both the first version of the SVDS and the final version of the SVDS demonstrated large positive correlations with self-declared SVD (*r*>0.5), indicating satisfactory convergent validity.

**Table 2:** Correlations between the short-video dependence scale and validation constructs.

Variable	SVDS^a^ 1.0 (n=402)	SVDS 2.0 (n=369)
Self-declared SVD^b^	0.62	0.58
Frequency of daily use	0.72	0.80
DSM-5^c^	0.68	0.73
CIAS^d^	0.53	0.61

^a^SVDS: short-video dependence scale.

^b^SVD: short-video dependence.

^c^DSM-5: Diagnostic and Statistical Manual of Mental Disorders, Fifth Edition.

^d^CIAS: Chinese Internet Addiction Scale.

### CFA and Internal Consistency of the Final Version of the SVDS Using a Massive Sample

Similar to the former round of CFA conducted on the third version of the SVDS, a model diagram was constructed using the 20 items and 4 factors. Model fit indices, including RMSEA, CFI, NFI, IFI, and TLI, were employed. Fortunately, the results obtained with the large sample were better than the results of the last CFA. Specifically, the results of the goodness-of-fit analysis were as follows: RMSEA=0.065; CFI=0.928; NFI=0.926; IFI=0.928; and TLI=0.916. All the data met the acceptable fit index criteria. Moreover, the internal consistency α coefficient of the 20 items was .93. The final version of the SVDS is presented in Table 3. The table shows the results after EFA and CFA, with the items rearranged according to the size of factor loading. Factor loading less than 0.4 has not been shown.

**Table 3:** The final version of the short-video dependence scale.

Index	Item	Dimension
1	Even if advised to watch fewer short videos, I find it difficult to do so.	Preoccupation and persistence
2	In my spare time, I don’t know what to do other than watching short videos.	Preoccupation and persistence
3	If I go without watching short videos for a long time, I fear missing out on popular videos or news.	Preoccupation and persistence
4	Even if I didn’t plan to watch short videos, I would subconsciously open the app.	Preoccupation and persistence
5	While studying or working, I often think about watching short videos.	Preoccupation and persistence
6	I have tried to spend less time watching short videos, but I can’t seem to do it.	Withdrawal and tolerance
7	Compared to last year, I spend more time watching short videos every day.	Withdrawal and tolerance
8	When I try to watch fewer videos, I feel bored or agitated.	Withdrawal and tolerance
9	My life seems uninteresting without short videos.	Withdrawal and tolerance
10	If asked to refrain from watching short videos for a week, I would find it difficult to resist the urge.	Withdrawal and tolerance
11	Watching short videos has had a negative impact on my academic or work performance.	Damage and hindrance
12	It has also negatively affected my physical health, such as eye strain and staying up late.	Damage and hindrance
13	I feel a decreased sense of self-worth due to my inability to control my time spent watching short videos.	Damage and hindrance
14	After watching short videos, I find it harder to focus on self-improvement.	Damage and hindrance
15	Even if I want to go to bed early, I still can’t resist watching short videos.	Damage and hindrance
16	When I watch short videos, it seems as if all of life’s problems disappear.	Deception and escape
17	I have tried to hide the negative effects that watching short videos has had on me from others.	Deception and escape
18	I tend to downplay my short-video viewing time when asked.	Deception and escape
19	After watching short videos, I tend to interact less with family and friends in real life.	Deception and escape
20	The actual amount of time I spend watching short videos is more than I realize.	Deception and escape

### Sensitivity and Specificity of the SVDS

The SVDS dimensions were evaluated for sensitivity and specificity. Sensitivity was determined based on positive responses from dependent short-video users in sample 2 (n=107), sample 3 (n=74), and sample 4 (n=16,038), while specificity relied on negative responses from nondependent short-video users in each sample. High sensitivity and specificity are ideal for effective discrimination of false positives and false negatives [44].

Initially, the first SVDS version demonstrated high sensitivity and sufficient specificity in sample 2. Corrected sensitivity and specificity values for each dimension were as follows: preoccupation, 0.87 and 0.45, respectively; tolerance, 0.96 and 0.44, respectively; withdrawal, 0.66 and 0.89, respectively; persistence, 0.88 and 0.61, respectively; escape, 0.98 and 0.32, respectively; problems, 0.85 and 0.84, respectively; deception, 0.68 and 0.85, respectively; and displacement, 0.58 and 0.93, respectively. In sample 4, the final SVDS version showed high sensitivity and sufficient specificity. Sensitivity and specificity values for each dimension were as follows: withdrawal and tolerance, 0.77 and 0.75, respectively; preoccupation and persistence, 0.83 and 0.87, respectively; impairment and hindrance, 0.87 and 0.80, respectively; and deception and escape, 0.62 and 0.79, respectively. Overall, the final SVDS version demonstrated stable sensitivity and specificity, with highly comparable diagnostic accuracy across the samples.

### Determination of the Clinical Cutoff Point for the Final Version of the SVDS

According to the SVDS modified based on the IAS in DSM-5, 4693 of the 16,038 participants were identified as dependent short-video users. This clinically diagnosed group was used as the gold standard. The diagnostic efficiency was demonstrated by a high AUC (0.83, 95% CI 0.95-1.00; *P*<.001).

Table 4 shows the sensitivity, specificity, 95% CI, PPV, and NPV of possible cutoff scores of the SVDS. At a score of 58, the sensitivity was 88.6% and specificity was 96.5%, and the Youden Index achieved its maximum value (85.2%). In this case, the prevalence of dependent short-video users was 4079 out of 16,038 (25.4%). The dependent short-video user group included 53.6% (2188/4079) males and 46.4% (1891/4079) females, while the nondependent short-video user group included 50.1% (5999/11,959) males and 49.8% (5960/11,959) females.

**Table 4:** Cutoff points for the short-video dependence scale based on clinical diagnostic interviews (N=16,038).

Criterion	Sensitivity^a^, value (95% CI)	Specificity^b^, value (95% CI)	Youden Index^c^	PPR^d^	NPR^e^
>54	89.58 (87.4-91.5)	86.50 (86.3-87.8)	0.7608	0.79	1.00
>55	89.47 (87.3-91.4)	89.90 (88.5-90.9)	0.7937	0.79	1.00
>56	89.47 (87.3-91.4)	92.68 (91.8-93.0)	0.8215	0.83	1.00
>57	89.47 (87.3-91.4)	95.43 (94.3-96.4)	0.8490	0.86	1.00
>58	88.61 (86.4-90.6)	96.54 (95.5-96.4)	0.8515	0.88	0.99
>59	87.22 (84.9-89.3)	97.41 (96.6-97.4)	0.8463	0.92	0.99
>60	86.40 (84.6-89.0)	97.75 (97.6-98.3)	0.8415	0.93	0.98
>61	85.07 (84.1-88.6)	98.76 (98.5-99.0)	0.8383	0.94	0.94
>62	83.57 (81.0-85.9)	99.03 (99.3-99.7)	0.8260	0.94	0.89

^a^Sensitivity: true negative/true negative and false positive.

^b^Specificity: true positive/true positive and false negative.

^c^Youden index: defined as sensitivity + specificity – 1.

^d^PPR: positive predictive rate (true positive/true positive and false positive).

^e^NPR: negative predictive rate (true negative/true negative and false negative).

### LPA Results

**Figure 2:**
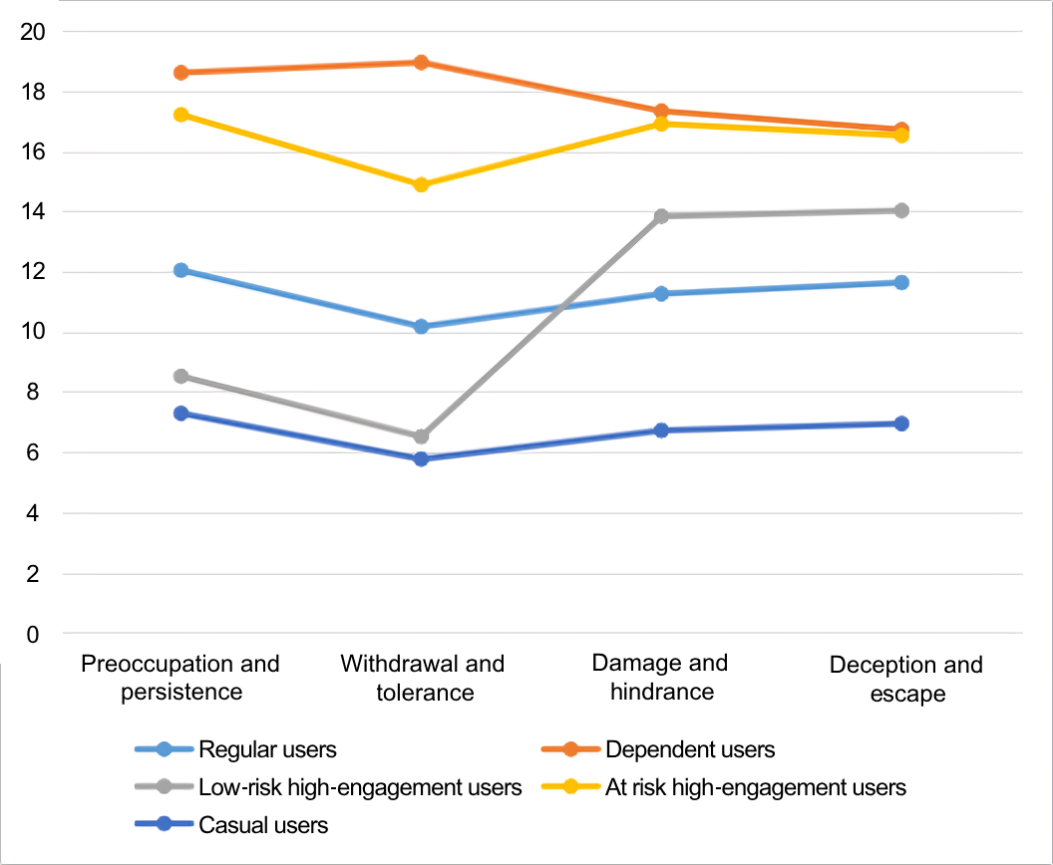
The 5 classes obtained from the latent profile analysis. The latent profile of short-video dependence based on short-video dependence scale (SVDS) scores. The X-axis represents the dimensions of the SVDS. The Y-axis represents the scores of each dimension of the SVDS.

The results of the LPA in the last sample are shown in Table 5. The AIC, BIC, and SSABIC values continued to decrease as the number of groups increased. The entropy was adequate for a 2-group solution to a 6-group solution. Despite the small difference in the data, the entropy of 5 classes was the smallest, indicating the highest degree of classification accuracy [45]; therefore, we selected the 5-group solution. The features of the 5 classes are shown in Figure 2. The first class, named “regular users” (6591/16,038, 41.1%), and the second class, named “casual users” (3224/16,038, 20.1%), represent adolescents who generally selected “very rarely” or “rarely” for all 20 items in the final version of the SVDS. The third class, named “low-risk high-engagement users” (2389/16,038, 14.9%), and the fourth class, named “at-risk high-engagement users” (2630/16,038, 16.4%), scored similarly higher on “damage and hindrance” and “deception and escape,” while the third class scored much lower on “preoccupation and persistence” and “withdrawal and tolerance” compared to the fourth class. The fifth class, named “dependent users” (1204/16,038, 7.5%), represents adolescents who generally rated their short-video use as “very often” or “often” for all 20 items in the scale.

**Table 5:** Results obtained from the latent profile analysis.

Model	Log-likelihood	Replicated log-likelihood	Free parameters, n	AIC^a^	BIC^b^	SSABIC^c^	Entropy	LMRT^d^
2 classes	–178532.773	Yes	13	357091.546	357191.894	357150.581	0.769	26,961.63
3 classes	–173702.934	Yes	18	347441.869	347580.812	347523.609	0.830	9464.91
4 classes	–171682.983	Yes	23	343411.967	343589.506	343516.413	0.861	3958.44
5 classes	–175612.864	Yes	28	341344.701	341560.835	341471.853	0.872	2035.38
6 classes	–169910.753	Yes	33	339887.506	340142.236	340037.364	0.808	1437.61

^a^AIC: Akaike information criterion.

^b^BIC: Bayesian information criterion.

^c^SSABIC: sample size–adjusted Bayesian information.

^d^LMRT: Lo-Mendell-Rubin adjusted likelihood ratio test.

## Discussion

### Principal Findings

This study aimed to develop and validate the SVDS, which is brief, is easy to administer, and has a clear diagnostic cutoff point to differentiate between dependent and highly engaged nondependent short-video users. Through item analysis, EFA, and CFA, the 20-item SVDS with 4 dimensions was established. To establish the diagnostic cutoff point, ROC curve analysis and LPA were conducted. Consequently, the final version of the SVDS with a clear cutoff point demonstrated strong internal consistency and convergent validity, and high sensitivity and specificity.

### Development Processes of the SVDS

We initially identified subjects with higher SVD through pretesting and in-depth interviews, extracting key dimensions and items from the raw data. We also used the 9 diagnostic criteria for IGD from the DSM-5 as a reference, which contributed to both the theoretical and conceptual development of the tool. This process led to the identification of 9 dimensions for the first version of the SVDS, each with 3 items. After item analysis, EFA, and CFA, a 20-item scale with 4 dimensions was finalized, resulting in 3 versions of the SVDS. Finally, using a large sample of 16,038 participants, the final version showed strong internal consistency and convergent validity.

### Final Version of the SVDS

The final (fourth) version of the SVDS included 4 dimensions: “withdrawal and tolerance,” “preoccupation and persistence,” “impairment and hindrance,” and “deception and escape.” Each dimension consisted of 5 items, and SVD was measured. Furthermore, we employed LPA and ROC curve analysis with a large sample of 16,038 people to detect dependent short-video users and define the cutoff point of 58 points. Five more subtypes of users of short videos were retrieved from the LPA.

### Final Version of the SVDS Showed Good Reliability and Validity

Our findings demonstrated that the final version of the SVDS is a reliable and valid measure of SVD. The CFA confirmed a good model fit, indicating strong external validity. Moreover, the final version of the SVDS exhibited satisfactory internal consistency (0.93) as well as good convergent validity. These results have important implications for identifying and preventing SVD and its potential negative consequences. Additionally, based on the diagnostic cutoff point of the final version of the SVDS, the prevalence of SVD in the last sample was 25.4% (4073/16,038).

Convergent validity was established by examining the CFA results of the SVDS and its correlations with similar constructs. The CFA results supported a good model fit for the final version of SVDS items, and as expected, there was a strong correlation between SVDS scores and CIAS scores.

### Final Version of the SVDS Showed Good Sensitivity and Specificity

Items of the final version of the SVDS exhibited good sensitivity and specificity. This indicates that the scale can accurately identify and differentiate between the presence and absence of social media dependence. Sensitivity refers to situations in which items correctly identify the target characteristic, while specificity refers to situations in which items correctly exclude the absence of the target characteristic. From the first version to the final version of the scale, there were improvements in its sensitivity and specificity, as outlined below.

### Confirmation of the Scale’s Dimensions

“Withdrawal and tolerance,” “preoccupation and persistence,” “impairment and hindrance,” and “deception and escape” were the 4 dimensions included in the SVDS.

First, withdrawal refers to discomfort when a substance or behavior is reduced or stopped, while tolerance means needing more to achieve the same effect. These are key addiction concepts, so individuals with high scores are closer to an addictive state and more likely to be “at-risk high-engagement users” or “dependent users.” Second, “preoccupation and persistence” involve constant thoughts about the activity and continuing despite harm, with higher scores reflecting stronger dependence. Third, “impairment and hindrance” refer to the negative impact on daily life and the reduced ability to resist addictive behavior. High scores suggest significant life problems and difficulty controlling behavior. Lastly, “deception and escape” involve lying or replacing one addictive behavior with another, with elevated scores indicating stronger dependence. The first 2 dimensions are core symptoms of SVD, as they directly impact the individual, while the latter 2 reflect the indirect effects of SVD on daily life.

### Establishment of the Diagnostic Cutoff Point

Using DSM-5–diagnosed dependent short-video users as the gold standard, this study suggests a clinically optimal cutoff score of 58 for diagnosing dependent short-video users with the final version of the SVDS. The best balance between sensitivity (88.6%) and specificity (96.5%) was achieved at this cutoff score. The LPA approach has been performed to identify cutoff points in the absence of a gold standard based on clinical interviews. The adequate diagnostic accuracy of the empirical cutoff point based on the LPA suggests that this approach is a reliable method to identify cutoff points.

We compared the SVDS scores of these dependent short-video users and established a SVDS cutoff point of 58, whereby individuals scoring equal to or higher than 58 were considered to be dependent short-video users.

### Comparison With Prior Work

#### Advantages Compared to DSM-5 Items for Behavioral Addictions

The symptoms of SVD are more closely aligned with the SVDS than with the DSM-5. Some of the parameters of the original SVDS indicated weak sensitivity or specificity because they were largely the same as those of the DSM-5. During the SVDS development process, we were able to improve the scale.

For the first version of the SVDS, the dimensions were identical to those of the DSM-5. Additionally, some items showed lower sensitivity and specificity. To start with, the items measuring displacement showed a lower sensitivity in comparison to the other items of the SVDS and the displacement item of the IGD scale [46]. This suggests that a few high short-video dependents lost interest in their previous hobbies and entertainment. This may be because short-video use can coexist with other activities to some extent. Existing studies in the literature have focused on internet addiction and media use, finding that people who are highly dependent on the internet may reduce offline activities and social interactions but do not necessarily lose interest in previous hobbies altogether [6,9,47]. This implies that people who are highly dependent on short videos may strike a certain balance between online and offline activities, rather than completely abandoning their previous hobbies.

Next, the items measuring escape, preoccupation, and tolerance showed a lower specificity in comparison to the other items. This suggests that most short-video users often use short videos to escape from negative feelings, irrespective of whether they are dependent or nondependent short-video users. This may be because short videos are often light-hearted and entertaining in nature, providing a pleasurable and entertaining experience. For low-dependent individuals, watching short videos may be a relaxing and enjoyable way to spend time, rather than an addictive behavior. Short videos also provide a virtual world where people can temporarily escape from real-life problems, stresses, and challenges. Watching short videos can be an escape mechanism that helps people temporarily avoid unpleasant or difficult situations.

#### Advantages Compared to Other Similar Questionnaires and Scales

We compared the SVDS to related scales (ie, measurement tools that include internet, dependence, addiction, or other similar keywords), such as the CIAS and the Social Media Disorder Scale (SMDS).

The CIAS is a scale used to assess the core symptoms and problems associated with internet addiction in the Chinese population [24,39]. It does not consider the different types or forms of internet addiction, such as social media addiction or short-form video addiction. Furthermore, it appears inappropriate to use a scale created nearly 20 years ago to gauge how dependent modern society is on short films. Therefore, the SVDS with reference to the content of in-depth interviews conducted in 2023 is more relevant to SVD and can provide more accurate results. Most importantly, we provided statistical evidence for a precise diagnostic cutoff point.

The SMDS is a scale used to assess users’ motivation to use social media and the degree of addiction [7]. It does not consider users’ communication and interaction with other people or groups on social media platforms, such as liking, commenting, etc, and the psychological and social impacts of such communication and interaction on users. Besides, the scale has only 9 dimensions, which may not cover all aspects of social media addiction, such as motivation, frequency, and duration of social media use. As a result, the SVDS, which has more detailed questions and has questions that are pertinent to the features of short-video platforms, is better suited for measuring SVD.

### Limitations and Future Directions

While this study provides distinctive insights into the detection of dependent short-video users and the prevalence of SVD among Chinese university students, it is imperative to acknowledge the presence of certain limitations in the research. First, there may be sampling bias as this study recruited subjects on the NAODAO and TClab platforms and focused on universities in one city. As a significant majority of the participants in our sample had at least a college-level education, there was an imbalance concerning age and educational background. Despite previous studies demonstrating measurement invariance of some scales across education and gender [48-50], the imbalance in the number of participants of different genders or education levels may still have affected our results. To further test the SVDS, future research should collect a well-balanced sample. Additionally, since our sample was primarily drawn from Chinese university students, specifically those in Hangzhou, the findings may have limited generalizability to other cultural or demographic contexts. Future studies are needed to validate the scale in diverse cultural settings and populations. Second, according to recent reports from social surveys, a sizable number of short-video consumers are middle-aged and older [51,52]. The SVDS should be administered to this demographic in subsequent investigations. Third, the self-completion scale might have a social desirability effect. Future studies could cross-validate our results using other research methods (eg, field studies). Fourth, future research should focus on examining the relationship between the degree of short-video dependency and individual characteristics such as personality traits, self-esteem, impulsivity, academic performance or work performance, and other relevant factors. Finally, it is important to examine whether there are differences in SVD among different groups, which could help validate the reliability and validity of the current scale.

### Conclusions

We followed the pragmatic approach of developing and validating an administration tool to measure SVD, which enables the investigation of trends and developments in the prevalence of SVD during the current period of rapid changes in the short-video landscape. The final version of the SVDS showed high reliability and validity. It could be applied by subsequent researchers in longitudinal studies to investigate the mechanisms of SVD in terms of behavioral, cognitive, and affective influences, as well as to develop effective interventions to deal with the problem of SVD. The development and validation of a questionnaire with a diagnostic cutoff point are essential for understanding and monitoring SVD, facilitating relevant academic investigations, and informing policy development.

## Data Availability

The data are stored at our lab-based network attachment system [53] (ID: guests; PIN: dong@123.COM).

## Appendix

Multimedia Appendix 1 Details of participants.

Multimedia Appendix 2 Details of the interviewees.

Multimedia Appendix 3 In-depth interview questionnaire.

Multimedia Appendix 4 Short-video dependence scale 1.0.

Multimedia Appendix 5 Short-video dependence scale 2.0.

Multimedia Appendix 6 Short-video dependence scale 3.0.

Multimedia Appendix 7 Coding interview.

Multimedia Appendix 8 Factor analysis of short-video dependence scale 2.0.

Multimedia Appendix 9 Short-video dependence scale 4.0.

## Abbreviations

AIC: Akaike information criterion
AUC: area under the receiver operating characteristic curve
BIC: Bayesian information criterion
CFA: confirmatory factor analysis
CFI: comparative fit index
CIAS: Chinese Internet Addiction Scale
DSM-5: Diagnostic and Statistical Manual of Mental Disorders, Fifth Edition
EFA: exploratory factor analysis
IFI: iterative fit index
IGD: internet gaming disorder
KMO: Kaiser-Meyer-Olkin
LPA: latent profile analysis
NFI: normed fit index
PCA: principal component analysis
RMSEA: root mean square error of approximation
ROC: receiver operating characteristic
SMDS: Social Media Disorder Scale
SSABIC: sample size–adjusted Bayesian information criterion
SVD: short-video dependence
SVDS: short-video dependence scale
TLI: Tucker-Lewis Index
