# Supplementary material for: Assessing Short-Video Dependence for e-Mental Health: Development and Validation Study of the Short-Video Dependence Scale
Source: J Med Internet Res. 2025 Mar 4;27:e66341. doi: 10.2196/66341 (PMC11920665; doi:10.2196/66341)
Supplement: Multimedia Appendix 1 [file jmir_v27i1e66341_app1.docx]

**Multimedia Appendix 1.** Details of participants.

In the first pretest conducted in October 2022, a total of 457 valid questionnaires were obtained. The participants' age ranged from 16 to 57 years, with an average age of 23.67 years and a standard deviation of 6.84 years. Among the participants, 39% were females.

One month later, in order to test the reliability and validity of the the first version of SVDS and explore the questionnaire's cut-off point, the second survey was conducted. Each participant completed three questionnaires, namely SVDS, CIAS, and DSM-5. The valid sample of DSM-5 were obtained from 490 participants aged 17 to 55 years, with an average age of 26.24 years and a standard deviation of 7.35 years. The number of questionnaires that met the criteria for convergent validity was 402 * 3 (SVDS, CIAS, and DSM-5), conducted among participants aged 17 to 45 years, with an average age of 24.97 years and a standard deviation of 7.21 years. Among them, 44% were females. In the process of screening valid questionnaires, we excluded questionnaires with a completion time of more than 2 standard deviations above the mean and questionnaires that did not pass the lie detection items.

Last but not least, to test the refined the second version of SVDS, the third survey was conducted two months later with a new set of participants. The participants completed SVDS, CIAS, and DSM-5 questionnaires. The valid sample was 369 * 3, with ages ranging from 17 to 63 years. The final version (second version) of the scale with 20 items was administered. The participants had an average age of 27.05 years and a standard deviation of 8.18 years. Among them, 46% were females.

Finally, a large sample of 16038 participants from HangZhou Normal University and Zhejiang University of Water Resources and Electric Power were included. The participants had an average age of 19.86 years and a standard deviation of 2.144 years. Among them, 48% were females.

All participants in this study received a small monetary reward. Data processing and analysis were performed using SPSS 27 for Windows and Amos 24. SPSS 27 was used for item analysis and exploratory factor analysis (EFA), while Amos 24 was used for confirmatory factor analysis (CFA). Additionally, scoring for the DSM-5 scale was based on a binary scoring system (yes = 1, no = 0), while both SVDS and CIAS were scored on a 5-point scale ranging from "none" to "severe," with scores ranging from 0 to 4 incrementally.
