# Supplementary material for: Assessing Short-Video Dependence for e-Mental Health: Development and Validation Study of the Short-Video Dependence Scale
Source: J Med Internet Res. 2025 Mar 4;27:e66341. doi: 10.2196/66341 (PMC11920665; doi:10.2196/66341)
Supplement: Multimedia Appendix 2 [file jmir_v27i1e66341_app2.docx]

## Multimedia Appendix 2. Details of the interviewees.

| \| **Table S1 Demographic information of participants from in-depth interview** \| \| \| \| \| \| \| \| \| \| \| --- \| --- \| --- \| --- \| --- \| --- \| --- \| --- \| --- \| --- \| \| **N** \| **Age min** \| **Age max** \| **University graduation rate** \| **Provinces** \| **Daily frequency more than 4h** \| **Self-declared dependence score over 4** \| **Dependence perceived by other people over 4** \| **Frequently liking and commenting** \| **Frequently downloading and adding to favorite** \| \| 15 \| 19 \| 27 \| 40% \| 12 \| 100% \| 53% \| 67% \| 100% \| 87% \| \|  \|  \|  \|  \|  \|  \|  \|  \|  \|  \|   **Table S2 Detail demographic information of participants from in-depth interview** | | | | | | | | |  |  |
| --- | --- | --- | --- | --- | --- | --- | --- | --- | --- | --- | --- | --- | --- | --- | --- | --- | --- | --- | --- | --- | --- | --- | --- | --- | --- | --- | --- | --- | --- | --- | --- | --- | --- | --- | --- | --- | --- | --- | --- | --- | --- | --- | --- | --- | --- | --- | --- | --- | --- | --- |
| Index | Age |  | State | Province | Daily frequence | Self-declared dependence | Dependence perceived by people around you | Frequently liking and commenting | Frequently downloading and adding to favorite | Doing things on short video platforms other than watching |
| **M1** | 21 |  | Working | ShangHai | more than 4h | 4 | 3 | yes | no | yes |
| **M2** | 22 |  | Working | ShanXi | more than 4h | 3 | 3 | yes | yes | yes |
| **F3** | 26 |  | Working | ShanDong | more than 4h | 3 | 3 | yes | yes | yes |
| **M4** | 19 |  | Schooling | ShanDong | more than 4h | 3 | 3 | yes | yes | yes |
| **M5** | 21 |  | Schooling | HeBei | more than 4h | 4 | 4 | yes | yes | yes |
| **M5** | 20 |  | Schooling | GuangDong | more than 4h | 4 | 4 | yes | yes | yes |
| **M7** | 27 |  | Working | JiangSu | more than 4h | 4 | 4 | yes | yes | yes |
| **F8** | 19 |  | Schooling | HuNan | more than 4h | 3 | 3 | yes | yes | yes |
| **M9** | 22 |  | Working | GuangDong | more than 4h | 4 | 4 | yes | yes | yes |
| **M10** | 26 |  | Working | HeNan | more than 4h | 3 | 3 | yes | yes | yes |
| **M11** | 19 |  | Schooling | HuBei | more than 4h | 3 | 3 | yes | yes | yes |
| **M12** | 20 |  | Schooling | ZheJiang | more than 4h | 4 | 4 | yes | no | yes |
| **M13** | 20 |  | Schooling | JiangXi | more than 4h | 4 | 3 | yes | yes | yes |
| **F14** | 20 |  | Schooling | ShanXi | more than 4h | 4 | 3 | yes | yes | yes |
| **M15** | 20 |  | Schooling | SiChuan | more than 4h | 3 | 3 | yes | yes | yes |
