# Supplementary material for: Assessing Short-Video Dependence for e-Mental Health: Development and Validation Study of the Short-Video Dependence Scale
Source: J Med Internet Res. 2025 Mar 4;27:e66341. doi: 10.2196/66341 (PMC11920665; doi:10.2196/66341)
Supplement: Multimedia Appendix 3 [file jmir_v27i1e66341_app3.docx]

## Multimedia Appendix 3. In-depth interview questionnaire.

1. When did you first start using short video platforms?
2. How often do you use it, and for what duration?
3. Rate your satisfaction with short video platforms usage on a scale of 1 to 10.
4. On a scale of 1 to 10, how would you rate your own dependence on short video platforms?
5. What types of content do you like to watch on short video platforms?
6. Have you ever felt time flying when using TikTok?
7. Do you repeatedly uninstall or temporarily stop using short video platforms?
8. What motivates you to use short video platforms?
9. Have you experienced any physical discomfort such as eye strain or sleep disturbances or any other physical discomfort due to usage of short video platforms?
10. What are your views pertaining to the use of big data for personalized video recommendations on short video platforms?
11. Apart from watching videos, have you used any other features on short video platforms, such as creating videos, shopping, live streaming, selling items, or local networking? Why do you choose to use these features on short video platforms instead of other apps?
12. Has anyone ever told you that you watch short video platforms too much? What do you think makes them feel that you watch it excessively?
