# Supplementary material for: Assessing Short-Video Dependence for e-Mental Health: Development and Validation Study of the Short-Video Dependence Scale
Source: J Med Internet Res. 2025 Mar 4;27:e66341. doi: 10.2196/66341 (PMC11920665; doi:10.2196/66341)
Supplement: Multimedia Appendix 4 [file jmir_v27i1e66341_app4.docx]

## Multimedia Appendix 4. Short-video dependence scale 1.0.

Short Video Dependence Scale

This scale is intended solely for scientific research purposes and all responses will be kept strictly confidential.

The following statements describe certain psychological states of individuals, and the items are neither right nor wrong. Please use a scale of 1 to 5 to indicate the extent to which each statement reflects your situation. Please respond to each item individually, without omission. Your participation is greatly appreciated.

To begin, answer the following questions by using this scale:

| 1 | Does not apply |
| --- | --- |
| 2 | Rarely |
| 3 | Occasionally |
| 4 | Often |
| 5 | Always |

|  | Question | Scale | | | | |
| --- | --- | --- | --- | --- | --- | --- |
| 1 | When studying or working, I often think about watching short videos. | 1 | 2 | 3 | 4 | 5 |
| 2 | If I haven't watched short videos for a while, I fear missing out on popular short videos or messages. | 1 | 2 | 3 | 4 | 5 |
| 3 | During my free time, I'm unsure of other activities to do if I don't watch short videos. | 1 | 2 | 3 | 4 | 5 |
| 4 | When I open the short video app, I initially plan to watch for a short while but end up watching for a long time. | 1 | 2 | 3 | 4 | 5 |
| 5 | Even if others suggest I watch short videos less, I find it challenging to do so. | 1 | 2 | 3 | 4 | 5 |
| 6 | Even without the intention to watch short videos, I subconsciously open the short video app. | 1 | 2 | 3 | 4 | 5 |
| 7 | Compared to last year, I spend more time watching short videos every day. | 1 | 2 | 3 | 4 | 5 |
| 8 | I have attempted to spend less time watching short videos but couldn't succeed. | 1 | 2 | 3 | 4 | 5 |
| 9 | When I try to stop watching and start doing something else, I can't resist reopening the app to continue watching. | 1 | 2 | 3 | 4 | 5 |
| 10 | When I attempt to watch fewer short videos, I feel bored or restless. | 1 | 2 | 3 | 4 | 5 |
| 11 | If I were required not to watch short videos for a week, I would find it hard to suppress the urge to watch. | 1 | 2 | 3 | 4 | 5 |
| 12 | My life seems devoid of joy without short videos. | 1 | 2 | 3 | 4 | 5 |
| 13 | While watching short videos, it feels like all my life's troubles disappear. | 1 | 2 | 3 | 4 | 5 |
| 14 | After watching short videos, my interactions with family or friends in real life decrease. | 1 | 2 | 3 | 4 | 5 |
| 15 | During my free time, I have many other things I can do besides watching short videos. | 1 | 2 | 3 | 4 | 5 |
| 16 | I enjoy interacting with strangers or friends on short video apps and look forward to their responses. | 1 | 2 | 3 | 4 | 5 |
| 17 | I have attempted to uninstall or temporarily stop using TikTok. | 1 | 2 | 3 | 4 | 5 |
| 18 | Even when I want to go to bed early, I can't help but watch short videos. | 1 | 2 | 3 | 4 | 5 |
| 19 | After watching short videos, it's more challenging for me to focus on self-improvement activities. | 1 | 2 | 3 | 4 | 5 |
| 20 | Watching short videos has negatively impacted my academic or work performance, such as late submissions or project delays. | 1 | 2 | 3 | 4 | 5 |
| 21 | Watching short videos has had a negative impact on my physical health, such as eye strain, staying up late, daytime fatigue, or discomfort in my neck and back. | 1 | 2 | 3 | 4 | 5 |
| 22 | I experience a decrease in self-esteem because I find it challenging to control the time spent watching short videos, leading to feelings of inadequacy. | 1 | 2 | 3 | 4 | 5 |
| 23 | When people around me ask how much time I spend watching short videos, I try to downplay the duration. | 1 | 2 | 3 | 4 | 5 |
| 24 | The actual time I spend watching short videos is more than what I initially thought. | 1 | 2 | 3 | 4 | 5 |
| 25 | Compared to last year, I spend less time watching short videos every day. | 1 | 2 | 3 | 4 | 5 |
| 26 | I have tried to hide the negative effects that watching short videos has on me from others. | 1 | 2 | 3 | 4 | 5 |
| 27 | During my free time, I barely have other things I can do besides watching short videos. | 1 | 2 | 3 | 4 | 5 |
