# Supplementary material for: Assessing Short-Video Dependence for e-Mental Health: Development and Validation Study of the Short-Video Dependence Scale
Source: J Med Internet Res. 2025 Mar 4;27:e66341. doi: 10.2196/66341 (PMC11920665; doi:10.2196/66341)
Supplement: Multimedia Appendix 6 [file jmir_v27i1e66341_app6.docx]

## Multimedia Appendix 6. Short-video dependence scale 3.0.

Short Video Dependence Scale

This scale is intended solely for scientific research purposes and all responses will be kept strictly confidential.

The following statements describe certain psychological states of individuals, and the items are neither right nor wrong. Please use a scale of 1 to 5 to indicate the extent to which each statement reflects your situation. Please respond to each item individually, without omission. Your participation is greatly appreciated.

To begin, answer the following questions by using this scale:

| 1 | Does not apply |
| --- | --- |
| 2 | Rarely |
| 3 | Occasionally |
| 4 | Often |
| 5 | Always |

|  | Question | Scale | | | | |
| --- | --- | --- | --- | --- | --- | --- |
| 1 | Even if advised to watch fewer short videos, I find it difficult to do so. | 1 | 2 | 3 | 4 | 5 |
| 2 | In my spare time, I don't know what to do other than watching short videos. | 1 | 2 | 3 | 4 | 5 |
| 3 | If I go without watching short videos for a long time, I fear missing out on popular videos or news. | 1 | 2 | 3 | 4 | 5 |
| 4 | Even if I didn't plan to watch short videos, I would subconsciously open the app. | 1 | 2 | 3 | 4 | 5 |
| 5 | While studying or working, I often think about watching short videos. | 1 | 2 | 3 | 4 | 5 |
| 6 | I have tried to spend less time watching short videos, but I can't seem to do it. | 1 | 2 | 3 | 4 | 5 |
| 7 | Compared to last year, I spend more time watching short videos every day. | 1 | 2 | 3 | 4 | 5 |
| 8 | When I try to watch fewer videos, I feel bored or agitated. | 1 | 2 | 3 | 4 | 5 |
| 9 | My life seems uninteresting without short videos. | 1 | 2 | 3 | 4 | 5 |
| 10 | If asked to refrain from watching short videos for a week, I would find it difficult to resist the urge. | 1 | 2 | 3 | 4 | 5 |
| 11 | Watching short videos has had a negative impact on my academic or work performance. | 1 | 2 | 3 | 4 | 5 |
| 12 | It has also negatively affected my physical health, such as eye strain, staying up late. | 1 | 2 | 3 | 4 | 5 |
| 13 | I feel a decreased sense of self-worth due to my inability to control my time spent watching short videos. | 1 | 2 | 3 | 4 | 5 |
| 14 | After watching short videos, I find it harder to focus on self-improvement. | 1 | 2 | 3 | 4 | 5 |
| 16 | Even if I want to go to bed early, I still can't resist watching short videos. | 1 | 2 | 3 | 4 | 5 |
| 17 | When I watch short videos, it seems as if all of life's problems disappear. | 1 | 2 | 3 | 4 | 5 |
| 18 | I have tried to hide the negative effects that watching short videos has had on me from others. | 1 | 2 | 3 | 4 | 5 |
| 19 | I tend to downplay my short video viewing time when asked. | 1 | 2 | 3 | 4 | 5 |
| 20 | After watching short videos, I tend to interact less with family and friends in real life. | 1 | 2 | 3 | 4 | 5 |
| 21 | The actual amount of time I spend watching short videos is more than I realize. | 1 | 2 | 3 | 4 | 5 |
