# Supplementary material for: Assessing Short-Video Dependence for e-Mental Health: Development and Validation Study of the Short-Video Dependence Scale
Source: J Med Internet Res. 2025 Mar 4;27:e66341. doi: 10.2196/66341 (PMC11920665; doi:10.2196/66341)
Supplement: Multimedia Appendix 7 [file jmir_v27i1e66341_app7.docx]

## Multimedia Appendix 7. Coding interview.

|  | | |
| --- | --- | --- |
| **Index** | **Organized interview content** | **Symptoms Keywords** |
| M1 | Usually do not particularly need, but free time in addition to Shake is WeChat (Displacement, Persistence); the more I watch, the more I want to watch (Tolerance); will be on it to buy things, shoot videos, and will also be on it to learn (Displacement); obviously will be watching videos because of eye soreness, reduced ability to concentrate, cervical and lumbar spine discomfort, and will also make my knowledge base narrow. Restlessness, lack of concentration, and vicious cycle (Problem). | Displacement, Persistence, Tolerance, Problem |
| M2 | Have uninstalled and reinstalled TikTok several times, can't restrain it (Withdrawal); obviously will stay up late because of watching videos, affecting rest, the more sleep the later (Problem, Tolerance, persistence). | Withdrawal, Problem, Tolerance, Persistence |
| F3 | Makes me procrastinate more (Problem, Escape). | Problem, Escape |
| M4 | Watching 6-8 hours a day (Preoccupation, Persistence); classmates around me are playing, can't play with them if I don't (Displacement). | Preoccupation, Persistence, Displacement |
| M5 | Will use the same-city dating function on it (Displacement); have been cheated when buying things on it and don't dare to tell my family (Deception, Problem). | Displacement, Deception, Problem |
| M5 | Watching it for 5-8 hours a day, feeling that "10 minutes on TikTok is an hour on earth" (Preoccupation, Persistence). | Preoccupation, Persistence |
| M7 | I will open a live broadcast on it, sell things, and buy things (Displacement); watching videos makes my eyes dry, and I have dark circles all the time (Problem); my family and friends have told me many times to brush less, but it's hard to control it (Preoccupation, Escape, Problem, Tolerance). | Displacement, Problem, Preoccupation, Escape, Tolerance |
| F8 | I don't know what to do in my free time without watching. And sent the video will be very concerned about the audience's feedback (Displacement); very hot, everyone is using it, swiping video seems to be something young people should do, people around me have shown that they feel they are swiping too much (Preoccupation, Persistence); watching too much will make my eyes sore, see things will become blurred, rest for a while and then it will be fine. Sometimes do things all the time look at the phone , procrastination, occasionally late homework (Problem). | Displacement, Preoccupation, Persistence, Problem |
| M9 | Look at more than 5 hours a day, a wake up will brush, easy to indulge, lose the concept of time, people around have shown that they feel that they brush too much, in the restraint, but it is difficult (Preoccupation, sustained); fragmented time are watching (Displacement, Preoccupation); selling things on it is very convenient, the quality can be, specialized shopping platforms do not have the entertainment. Will also make friends in the same city on it, specialized social media are full of acquaintances and inconvenient. Will be on it to obtain knowledge, to understand current events. Will feel a sense of presence when receiving video likes and comments. (Displacement); Push ads are too precise, there is a feeling of being spied on privacy, watching too much will cause whiplash (Problem). | Preoccupation, Persistence, Displacement, Problem |
| M10 | Watching more than 5 hours a day, people around me have shown that they feel like they brush too much (Preoccupation, Persistence). | Preoccupation, Persistence |
| M11 | Watching all the fragmented time, 4-5 hours a day (Preoccupation, Persistence); not brushing Shake it will be a little uncomfortable (Withdrawal); neck forward, sore eyes, roommates and parents show that they feel they brush too much (Problem). | Preoccupation, Persistence, Withdrawal, Problem |
| M12 | Swipe almost 7 hours a day (Preoccupation, Persistence); have things to do, but it's hard to stop at any time, it's hard to stop, need to do a mental struggle (Withdrawal, Problem); haven't uninstalled, Shakeology is very important to me; eyes are sore, back ache, neck ache, sleep is affected, will be swiping until 3 or 4 o'clock (Problem). | Preoccupation, Persistence, Withdrawal, Problem |
| M13 | Watch 3-5h a day, brush when I'm bored, can't always stop even if I want to, watching live streaming delays things, watching short videos will want to finish it, procrastinate doing things (Preoccupation, Withdrawal); tried to uninstall it during the exam week, but failed, will continue to brush. Sore eyes, back pain and neck pain, will affect sleep, will go to bed late (Problem); classmates and parents think I watch too much, but it's hard to change, still watch for a long time (Escape). | Preoccupation, Withdrawal, Problem, Escape |
| F14 | Watching 3-6h a day, not class time are watching, watching more on weekends (Preoccupation); a long time without watching will be twisted, feel like there is nothing to do (Withdrawal, replacement); before the exam have uninstalled seven or eight times, before going to bed to brush for a long time, the neck and eyes will be uncomfortable, stay up late, go to bed two or three hours later than usual (Problem). | Preoccupation, Withdrawal, Displacement, Problem |
| M15 | TikTok is very practical ah, the search bar looks more convenient than Baidu kind of text version, and then also fast access to knowledge, you can share with friends (Displacement). | Displacement |
