# Supplementary material for: Assessing Short-Video Dependence for e-Mental Health: Development and Validation Study of the Short-Video Dependence Scale
Source: J Med Internet Res. 2025 Mar 4;27:e66341. doi: 10.2196/66341 (PMC11920665; doi:10.2196/66341)
Supplement: Multimedia Appendix 8 [file jmir_v27i1e66341_app8.docx]

## Multimedia Appendix 8. Factor analysis of short-video dependence scale 2.0.

|  | | | | | | | | |  |
| --- | --- | --- | --- | --- | --- | --- | --- | --- | --- |
| **Index** | **Item** | **Factor loading** | | | | | | **Communalities** |  |
|  |  | 1 | 2 | 3 | | 4 | |  |  |
| 5 | Even if advised to watch fewer short videos, I find it difficult to do so. | 0.698 |  |  |  | | 0.621 | | |
| 3 | In my spare time, I don't know what to do other than watching short videos. | 0.697 |  |  |  | | 0.604 | | |
| 2 | If I go without watching short videos for a long time, I fear missing out on popular videos or news. | 0.648 |  |  |  | | 0.534 | | |
| 6 | Even if I didn't plan to watch short videos, I would subconsciously open the app. | 0.613 |  |  |  | | 0.655 | | |
| 1 | While studying or working, I often think about watching short videos. | 0.6 |  |  |  | | 0.675 | | |
| 8 | I have tried to spend less time watching short videos, but I can't seem to do it. |  | 0.729 |  |  | | 0.669 | | |
| 7 | Compared to last year, I spend more time watching short videos every day. |  | 0.719 |  |  | | 0.627 | | |
| 10 | When I try to watch fewer videos, I feel bored or agitated. |  | 0.712 |  |  | | 0.66 | | |
| 12 | My life seems uninteresting without short videos. |  | 0.624 |  |  | | 0.668 | | |
| 11 | If asked to refrain from watching short videos for a week, I would find it difficult to resist the urge. |  | 0.597 |  | |  | | 0.634 | |
| 19 | Watching short videos has had a negative impact on my academic or work performance. |  |  | 0.78 | |  | | 0.642 | |
| 20 | It has also negatively affected my physical health, such as eye strain, staying up late. |  |  | 0.715 | |  | | 0.658 | |
| 21 | I feel a decreased sense of self-worth due to my inability to control my time spent watching short videos. |  |  | 0.655 | |  | | 0.68 | |
| 18 | After watching short videos, I find it harder to focus on self-improvement. |  |  | 0.651 | |  | | 0.583 | |
| 17 | Even if I want to go to bed early, I still can't resist watching short videos. |  |  | 0.516 | |  | | 0.508 | |
| 13 | When I watch short videos, it seems as if all of life's problems disappear. |  |  |  | | 0.638 | | 0.703 | |
| 24 | I have tried to hide the negative effects that watching short videos has had on me from others. |  |  |  | | 0.606 | | 0.684 | |
| 22 | I tend to downplay my short video viewing time when asked. |  |  |  | | 0.553 | | 0.658 | |
| 14 | After watching short videos, I tend to interact less with my family and friends in real life. |  |  |  | | 0.482 | | 0.498 | |
| 23 | The actual amount of time I spend watching short videos is more than I realize. |  |  |  | | 0.477 | | 0.53 | |
|  | Eigenvalues | 6.517 | 3.835 | 3.843 | | 2.043 | |  | |
|  | Contribution rate (%) | 20.066 | 14.749 | 14.78 | | 12.859 | |  | |
|  | Cumulative contribution rate (%) | 20.066 | 34.814 | 49.594 | | 62.456 | |  | |
